# *Mariapanteles* (Hymenoptera, Braconidae), a new genus of Neotropical microgastrine parasitoid wasp discovered through biodiversity inventory

**DOI:** 10.3897/zookeys.208.3326

**Published:** 2012-07-17

**Authors:** James B. Whitfield, José L. Fernández-Triana, Daniel H. Janzen, Winnie Hallwachs, M. Alex Smith, Sophie Cardinal

**Affiliations:** 1 Department of Entomology, 320 Morrill Hall, 505 S. Goodwin Ave., University of Illinois, Urbana, IL 61801 USA; 2 Department of Integrative Biology & the Biodiversity Institute of Ontario, University of Guelph, Guelph, Ontario, N1G 2W1 CANADA; 3 Canadian National Collection of Insects, Ottawa, ON K1A 0C6, CANADA; 4 Canadian National Collection of Insects, Ottawa, ON K1A 0C6, CANADA

**Keywords:** Microgastrinae, new genus, Area de Conservación Guanacaste, Costa Rica, Brazil, Neotropics, parasitoid wasp, rain forest

## Abstract

A new genus of microgastrine parasitoid wasps, *Mariapanteles* Whitfield & Fernández-Triana, **gen. n.**, is described from rain forests of the Neotropics. The new genus is related to the common and speciose genus *Pseudapanteles*, but can be distinguished from the latter by having a complete transverse carina on the propodeum which forks around the spiracles. A molecular analysis based on data from COI from specimens of the proposed new genus plus possibly related genera confirms its generic distinctness. A key to two known species, *Mariapanteles felipei* Whitfield, **sp. n.** (Costa Rica) and *Mariapanteles dapkeyae* Fernández-Triana, **sp. n.** (Brazil) is provided. Evidence from collections suggests that there are other undescribed Neotropical congenerics. Specimens of *Mariapanteles* were likely confused in the past with the genus *Beyarslania* (referred to as *Xenogaster* until recently) but present information suggests that *Beyarslania* is restricted to the Afrotropical region while the Neotropical species clearly belong to a different genus, which we propose as new.

## Introduction

In the past few decades, biodiversity inventories in the neotropics have begun to incorporate intensive rearing programs for caterpillars, their food plants, and their parasitoids. The most extensive of these programs is in the Area de Conservación de Guanacaste (ACG) in northwestern Costa Rica. ACG contains 130,000 terrestrial hectares of contiguous conserved dry forest, cloud forest, and rain forest, extending from the Pacific Ocean to 2,000 m elevation and then down into the Caribbean lowlands (Janzen 2000, Janzen and Hallwachs 2011). The caterpillar/plant/parasitoid inventory is one of the main components of ACG conservation (e.g. Janzen and Hallwachs 2011, 2012; Janzen et al. 2009; Smith et al. 2006, 2007, 2008; Sharkey et al. 2011; Burns et al. 2007, 2008; Schauff et al. 2001; Gauld and Janzen 2004). Microgastrinae have been one of the most intensively studied groups of ACG parasitoids (e.g., Valerio et al. 2009; Smith et al. 2008; Grinter et al. 2009; Deans et al. 2003, Whitfield et al. 2011), but many hundreds of species of these tiny wasps still remain to be discovered and described. Microgastrines are probably the single most speciose higher taxon of parasitoids of Lepidoptera (Whitfield 1995, 1997), and it is estimated that 800+ species occur in ACG alone (Janzen and Hallwachs, inventory in progress).

The ACG inventory has encountered a rain forest species of Microgastrinae that superficially resembles the rare Old World genus *Beyarslania* - widely known as *Xenogaster* until a recent nomenclatural change (Kocak and Kemal 2009). This genus is only known from a single described species from South Africa, but undescribed species have been thought to exist in the Neotropics (Whitfield 1997; Campos and Diego 2001). As we studied the ACG specimens and other similar Neotropical specimens, we realized that these undescribed species are more informatively placed in a new genus, which we erect and describe here along with descriptions of two species contained within it. A molecular phylogenetic analysis based on data from the COI (“barcoding”) gene is included to provide a further test of the monophyly of this putative new genus.

## Methods

The specimens for this study came from two main sources: the ACG inventory (Janzen and Hallwachs 2012, Janzen et al. 2009) and unsorted Neotropical wasps from the Canadian National Collection of Insects (CNC) in Ottawa. The genotype for the new genus has been deposited in the Smithsonian Institution (NMNH) and the remaining specimens have been deposited in the CNC, Illinois Natural History Survey (INHS) and the Natural History Museum, London (BMNH).

The morphological terms and morphological measurements follow mostly Wharton and Sharkey (1997) and Valerio and Whitfield (2009).

Photos were taken with a Keyence VHX-1000 Digital Microscope, using a lens with a range of 13–130×. Plates for the illustrations were prepared using Adobe Photoshop, but images were not digitally enhanced.

DNA barcodes for these and all other ACG inventory Microgastrinae were obtained using DNA extracts prepared from single legs using a glass fibre protocol (Ivanova et al. 2006). Extracts were re-suspended in 30 μl of dH2O, and a 658-bp region near the 5’ terminus of the COI gene was amplified using standard primers (LepF1–LepR1) following established protocols (Smith et al. 2006, 2007, 2008). If the initial 658 bp amplification was not successful composite sequences were generated using internal primers. Primer information for individuals sequences can be retrieved from the Barcode of Life Data System (BOLD) (Ratnasingham and Hebert 2007) using the accessions detailed in the online supplementary table , but primers are as detailed in Smith et al. (2008). Full details of methodology are as in (Smith et al. 2006, 2007, 2008). All sequence data are available on BOLD (www.barcodinglife.org ) in the public Dataset, “*Mariapanteles* (Hymenoptera: Braconidae), a new genus of Neotropical microgastrine parasitoid wasp discovered through biodiversity inventory”. All collection information, BOLD, and GenBank accessions for all sequences are listed in the Table in online supplementary materials.

All available DNA barcodes for *Pseudapanteles* (32 New World species, most of which are undescribed) and *Mariapanteles* (2 species) that were at least 300 bp longwere downloaded from BOLD (see the online supplementary table). DNA barcodes for selected species of *Prasmodon*, *Diolcogaster*, *Microplitis*, *Cotesia*, *Apanteles*, and *Neoclarkinella* were also downloaded to be used as outgroups in the phylogenetic analyses. Genbank accession numbers of all sequences used are given in Table 1. Prior to analysis, identical *Pseudapanteles* sequences were removed from the dataset so that each unique sequence was only represented once. Sequences were aligned in Geneious Pro 5.5.6 (Drummond et al. 2011) using default settings for MUSCLE alignment. The first character was deleted from the aligned matrix because most sequences were missing this character. Bayesian phylogenetic analysis was performed in MrBayes v.3.1.2 (Ronquist and Huelsenbeck 2003) through the CIPRES Science Gateway V.3.1 (Miller et al. 2010). Model selection was based on Bayesian Information Criteria as implemented in JModelTest v.0.1.1 (Posada 2008). Two independent analyses with 4 chains each were run in parallel for 10 million generations under a GTR+I+G model. The parameter trace files of each run were observed in Tracer v.1.5 (Rambaut and Drummond 2009) to verify that the runs had converged on the same stationary distribution, and to decide on the appropriate number of generations to discard as burn-in. A maximum clade credibility tree was constructed from these 18 million post-burn-in generations in TreeAnnotator v1.7.0 (Rambaut and Drummond 2012). The above protocol was followed for two additional analyses in which all 3^rd^ codon positions were removed from the dataset to correct for potential problems stemming from saturation in 3^rd^ codon positions of COI.

**Table 1.** Specimens included in the COI molecular analyses and their GenBank Accession numbers. Sample IDs are DHJPAR numbers (assigned to ACG specimens submitted to BOLD) or other IDs submitted to BOLD. More complete data on all specimens are included in Appendix 1.

Taxon	Sample ID	Genbank Accession Number
*Apanteles* Rodriguez03	DHJPAR0012802	JQ847281
*Apanteles* Rodriguez05	DHJPAR0012285	EU396474
*Apanteles* Rodriguez169	DHJPAR0038032	JQ848826
*Cotesia* Whitfield03	DHJPAR0013374	JQ848576
*Diolcogaster* Choi04	DHJPAR0004153	HQ549146
*Mariapanteles dapkeyae* F.-T.	CNCHYM 03387	JQ849377
*Mariapanteles felipei* Whitfield	DHJPAR0025453	HQ549955
*Mariapanteles felipei* Whitfield	DHJPAR0025443	JN282317
*Microplitis* Whitfield19	DHJPAR0031685	HQ548870
*Neoclarkinella* sp.jft10	WAM 0011	JQ852287
*Neoclarkinella* sp.jft11	GOU 0608	JQ849914
*Prasmodon* Whitfield02	DHJPAR0038222	HQ548705
*Prasmodon* Whitfield05	DHJPAR0012956	HQ548880
*Pseudapanteles gouleti* F.-T.	CAM 0874	JQ848150
*Pseudapanteles* sp. jft8	CNCHYM 03312	JQ849707
*Pseudapanteles* sp. jft16	CNCHYM 03343	JQ575645
*Pseudapanteles* sp. jft19	CNCHYM 03355	JQ850586
*Pseudapanteles* sp. jft23	CNCHYM 03372	JQ850275
*Pseudapanteles* sp. jft23	CNCHYM 03372	JQ850275
*Pseudapanteles* sp. jft23	CNCHYM 03369	JQ854346
*Pseudapanteles* sp. jft25	CNCHYM 03377	JQ853286
*Pseudapanteles* sp. jft25	CNCHYM 03377	JQ853286
*Pseudapanteles* sp.jft29	10BBHYM-1279	JQ852886
*Pseudapanteles* sp.jft29	Micro0094	JQ852261
*Pseudapanteles* sp.jft29	Micro0269	JQ850735
*Pseudapanteles* Whitfield01	DHJPAR0004755	JQ849938
*Pseudapanteles* Whitfield02	DHJPAR0025345	JQ850204
*Pseudapanteles* Whitfield05	DHJPAR0031341	JQ852695
*Pseudapanteles* Whitfield05	DHJPAR0026205	HQ549736
*Pseudapanteles* Whitfield05	DHJPAR0033906	JQ576585
*Pseudapanteles* Whitfield06	DHJPAR0031347	HQ930249
*Pseudapanteles* Whitfield06	DHJPAR0031191	JQ849350
*Pseudapanteles* Whitfield06	DHJPAR0034081	JQ853689
*Pseudapanteles* Whitfield07	DHJPAR0013217	JQ852406
*Pseudapanteles* Whitfield08	DHJPAR0026281	JQ849770
*Pseudapanteles* Whitfield09	DHJPAR0027627	JN281617
*Pseudapanteles* Whitfield09	DHJPAR0027692	HM430666
*Pseudapanteles* Whitfield09	DHJPAR0027392	JQ849832
*Pseudapanteles* Whitfield09	DHJPAR0026088	JQ854468
*Pseudapanteles* Whitfield09	DHJPAR0026012	JQ853711
*Pseudapanteles* Whitfield09	DHJPAR0026026	HQ549676
*Pseudapanteles* Whitfield09	DHJPAR0027661	JQ576067
*Pseudapanteles* Whitfield09	DHJPAR0033842	HQ550374
*Pseudapanteles* Whitfield09	DHJPAR0031297	JQ855464
*Pseudapanteles* Whitfield10	DHJPAR0012880	JQ854478
*Pseudapanteles* Whitfield11	DHJPAR0031742	JQ847036
*Pseudapanteles* Whitfield12	DHJPAR0025380	HQ550092
*Pseudapanteles* Whitfield14	DHJPAR0025854	HQ549888
*Pseudapanteles* Whitfield15	DHJPAR0025751	JQ849472
*Pseudapanteles* Whitfield17	DHJPAR0026060	JN281784
*Pseudapanteles* Whitfield18	DHJPAR0027669	JQ853458
*Pseudapanteles* Whitfield19	DHJPAR0027440	JQ848147
*Pseudapanteles* Whitfield19	DHJPAR0026033	JQ847890
*Pseudapanteles* Whitfield19	DHJPAR0026268	JQ852987
*Pseudapanteles* Whitfield19	DHJPAR0027440	JQ848147
*Pseudapanteles* Whitfield19	DHJPAR0026033	JQ847890
*Pseudapanteles* Whitfield19	DHJPAR0026268	JQ852987
*Pseudapanteles* Whitfield19	DHJPAR0025022	HQ926353
*Pseudapanteles* Whitfield19	DHJPAR0026008	JQ850045
*Pseudapanteles* Whitfield19	DHJPAR0025959	JQ849085
*Pseudapanteles* Whitfield19	DHJPAR0025866	JQ852050
*Pseudapanteles* Whitfield19	DHJPAR0026534	HQ550096
*Pseudapanteles* Whitfield19	DHJPAR0027150	HQ548886
*Pseudapanteles* Whitfield19	DHJPAR0025831	JQ574919
*Pseudapanteles* Whitfield20	DHJPAR0040493	JQ849256
*Pseudapanteles* Whitfield20	DHJPAR0027329	JQ848647
*Pseudapanteles* Whitfield20	DHJPAR0039680	JQ848927
*Pseudapanteles* Whitfield20	DHJPAR0041914	JQ847496
*Pseudapanteles* Whitfield21	DHJPAR0038402	EU396445
*Pseudapanteles* Whitfield21	DHJPAR0027221	JN282176
*Pseudapanteles* Whitfield23	DHJPAR0031749	JQ852705
*Pseudapanteles* Whitfield27	DHJPAR0043058	JQ850352

## Results

### Molecular confirmation of *Mariapanteles* monophyly

Evidence (beyond the morphologcial data presented below) for the separation of these two genera comes from DNA barcode data (Figs 13, 14). When comparing the two species of *Mariapanteles* to 32 species of *Pseudapanteles* (most of them undescribed) for which there are CO1 sequences available in BOLD (www.boldsystems.com ), the two genera are recovered as separate groups.

Bayesian analysis of the COI sequences, with and without 3^rd^ codon positions included, clearly supports *Mariapanteles* as being a separate distinct group from *Pseudapanteles* (Figs 13, 14). *Pseudapanteles* was recovered as being monophyletic with posterior probability (PP) of 1 when 3^rd^ codon positions were included and PP of 0.8 when 3^rd^ codon positions were removed. Monophyly of *Mariapanteles* was recovered with PP of 1 in both analyses. Furthermore, *Mariapanteles* was not recovered as the sister group to *Pseudapanteles* in either analysis. Several other genera included as potentially disrupting *Mariapanteles* monophyly also failed to do so in the analyses, so we have concluded that *Mariapanteles* is indeed a distinct genus.

#### Mariapanteles

Whitfield & Fernández-Triana gen. n.

urn:lsid:zoobank.org:act:917FB3C2-D102-4884-9FC4-787CC81AD5E9

http://species-id.net/wiki/Mariapanteles

Figs 1 –12

##### Type species.

*Mariapanteles felipei* Whitfield sp. n., by present designation.

##### Genus diagnosis.

Propodeum with a complete transverse carina that forks around spiracles and reaches the lateral margin of propodeum, where it intersects a raised lateral carina. Fore wing without areolet (veins r-m and 3RS absent). First mediotergite with a sharp median groove on the basal half. The only other genera of Neotropical microgastrines with a complete transverse carina on the propodeum, *Clarkinella* and *Prasmodon*, both lack a medial groove on the first mediotergite and have an areolet in the forewing (a small areolet in *Clarkinella*, a large and quadrangular one in *Prasmodon*). *Mariapanteles* resembles *Pseudapanteles* in fore wing venation, shape of mediotergites 1 and 2, and general appearance of the body. However, *Pseudapanteles* has an elongate, bifurcate glossa, lacks a complete transverse carina on the propodeum, and the hypopygium has a large translucent fold with many pleats; the glossa of *Mariapanteles* is not bifurcate and the hypopygium has a median translucent fold with no or only a few pleats visible.

##### Description.

Body length 2.4–2.6 mm, fore wing length 2.6–2.9 mm, antenna about the same length as body. Pronotum with two lateral grooves present, the lower one excavated. Mesoscutum more or less uniformly sculptured by impressed punctures. Mesoscutum 1.3–1.4× wider than long. Mesoscutum and scutellum uniformly covered by dense, pale yellowish pilosity. Scutellum length/width at base 1.0-1.1X. Scutellar suture broad, with 4–8 costulae. Posterior band of scutellum polished. Scutellar lateral face with the polished area thin (15–25% the face height) and about half the face width. Mesopleuron mostly smooth and glabrous, except for punctures on the anterior margin and setae on all margins. Metapleuron mostly smooth, with some punctures and setae in the apical half; metapleuron with a crenulate, longitudinal sulcus running from lower margin near metacoxa through spiracle. Metapleural carina raised, with a short lamella. Propodeum mostly smooth; median carina well defined and raised its entire length, and with a clearly complete transverse carina that reaches the spiracles and forks around them (there may also be additional, shorter transverse carinae, some of them radiating from the median carina but not reaching the spiracles). Transverse carina on propodeum delimiting two areas, the anterior, basal one being more or less horizontal; the posterior, apical one is declivous. Mediotergite 1 mostly smooth and with a deep medial groove on its basal half; slightly widening for the first quarter of its length, then narrowing towards apex. Mediotergite 2 mostly smooth, transverse, subtriangular to trapezoidal in shape. Mediotergite 3 and following, unsculptured, polished and with sparse setae. Hypopygium mostly inflexible but with a medial, translucent fold ventrally where none or few (1–2) pleats are distinguishable. Ovipositor sheaths fully setose, 0.7× as long as metatibia length. Metacoxa long, surpassing the length of the third metasomal tergite. Metatibial inner spur longer than outer spur, and about half the length of metatarsomere 1. Metafemur more than 3.0× as long as wide. Fore wing without an areolet, vein R1a longer than stigma length, and vein r and 2RS evenly curved to very slightly arched (with no clear limits between the two veins). Hind wing with edge of vannal lobe medially straight to slightly concave and with uniformly distributed setae that are shorter than those at base and apex of the lobe.

##### Distribution.

The genus occurs in Central and South American rain forests. We describe two new species, one from Costa Rica (ACG, from rain forest at 400m) and one from Brazil (Mato Grosso and Goiás; the localities are presumed to have been rain forests at the time the specimens were collected). The CNC collection contains two additional specimens from other areas of Brazil that may represent additional species, but because they are singletons we do not describe them here. It is likely that more species of this new genus will be found in Neotropical rain forests.

##### Biology.

Unknown. All specimens have been collected with Townes-type Malaise traps.

##### Etymology.

*Mariapanteles* is dedicated to María Marta Chavarría Díaz of ACG and San Jose, Costa Rica, in recognition of her 30+ years of dedication to Costa Rican conservation, biodiversity systematics, and biodiversity development throughout Costa Rica, and very specifically within Area de Conservación de Guanacaste.

##### Comments.

*Mariapanteles* is closely related to *Pseudapanteles*,and future revisions of the phylogeny of Microgastrinae might find that its erection renders *Pseudapanteles* paraphyletic. For example, and according to Mason (1981), some species of *Pseudapanteles* could have a multiple or indefinite transverse carina, in which case the complete transverse carina in *Mariapanteles* might be seen as the extreme in a continuum from having no transverse carina to having the complete transverse carina of *Mariapanteles*. However, we consider that the presence of a complete transverse carina on the propodeum, forking around the propodeal spiracles, may be a strong autapomorphy that defines *Mariapanteles*. There are only four other genera of Microgastrinae with a similar, complete transverse carina on the propodeum: *Beyarslania*, *Clarkinella*, *Neoclarkinella*, and *Prasmodon*. However, they all appear to be only distantly related to *Mariapanteles* because they all lack a sharp median groove on mediotergite 1 and/or have an areolet in the fore wing.

The described species of *Pseudapanteles* never have a complete transverse carina. Most of the specimens in collections just have a simple median carina, with only few species having irregular transverse striations arising along the length of the median carina (but even in those cases they never reach the spiracle and never form a fork around them). One example can be seen in the original description and pictures of the species *Pseudapanteles gouleti* Fernández-Triana, from Canada (Figure 18, page 24, in Fernández-Triana 2010). However, the carination pattern is not comparable to a complete transverse carina – as displayed by *Mariapanteles*.

The presence of a bifurcate glossa is a strong autapomorphy for *Pseudapanteles* (it is only present in three other distantly related Microgastrinae genera: *Napamus*, *Promicrogaster* and *Sendaphne*). Furthermore, the differences between *Mariapanteles* and *Pseudapanteles* with respect to the pleated area of the hypopygium are also consistent in the separation of these two genera.

Currently *Pseudapanteles* has nine described species (Yu et al. 2009), and a wide distribution within the New World, ranging from Canada to South America (one of the species has also been introduced to Hawaii (Coulson 1992)). The actual number of species is much higher, and we have seen in collections several dozen undescribed species, mostly from the Neotropics. The vast majority of those specimens are remarkably invariant in having a bifurcate glossa and in lacking a transverse carina on the propodeum.

For all of the above reasons along with the molecular results, we have decided that *Mariapanteles* is a distinct, separate genus that may be closely related to *Pseudapanteles*.

As for the former records of Neotropical species of the genus *Beyarslania* (at that time called *Xenogaster*) (Whitfield 1997; Campos 2001), these are based on confusion with specimens of what we have described here as *Mariapanteles*. Based on the available evidence, we now consider *Beyarslania* to be restricted to the African tropics. The Neotropical specimens thought to belong to that genus should be identified as *Mariapanteles*.

##### Key to *Mariapanteles* species described here

**Table:** 

1	Body colour mostly yellow in females and males —at most, males with tergite 3+ brown (Fig. 8); scutellum mostly smooth and scutellar suture with 4-6 costulae (Fig. 12); propodeum with a complete transverse carina that is clearly independent of other smaller transverse carinae (Figs 9, 12) [Mato Grosso and Goiás, Brazil]	*Mariapanteles dapkeyae* sp. n.
–	Body colour in females mostly orange-yellowish but with some areas brown or reddish brown (apical edge of scutellum, metascutellum, some carina on propodeum, central area on mediotergites 3+) (Fig. 1), male with much darker coloration, especially in the interocellar area, mesosoma and metasoma (Fig. 2); scutellum with impressed punctures and scutellar suture with 6-8 costulae (Fig. 6); propodeum with a completely transverse carinae not always clearly delimited from other smaller transverse carina (Fig. 6) [ACG, northwestern Costa Rica]	*Mariapanteles felipei* sp. n.

## Species descriptions

### Mariapanteles felipei

Whitfield sp. n.

urn:lsid:zoobank.org:act:2A5E1320-5C93-45FE-AEB0-AF9382929A78

http://species-id.net/wiki/Mariapanteles_felipei

Figs 1–6

#### Holotype.

**Female** (NMNH).COSTA RICA: Alajuela Province, Sector Rincon Rain Forest of ACG, Caribe, Rio Francia, 400 m, latitude 10.90093, longitude -85.28915; 11–17.vii.2007, Malaise Trap. Voucher code: DHJPAR0025453.

#### Paratype.

Male (NMNH).Same data as for holotype, except for collecting date: 22-28.viii.2007. Voucher code: DHJPAR0025443.

#### Description.

**Female.** Antenna about the same length as body; body length 2.6 mm; forewing 2.9 mm. Head: face with shallow and sparse punctures and sparse, uniformly distributed setae; face width at antennal base/face width at clypeus edge: 1.1×; intertentorial pit distance/face width at clypeus edge: 0.5×; compound eye height/head height: 0.8×; head height/width: 0.8×; face width at antennal base/head maximum width: 0.6×; malar space/basal width of mandible 1.3×; clypeus width/height: 3.1×. Length/width of flagellomeres: 2nd (2.3×), 8th (2.5×), 14th (1.3×). Length of flagellomere 2^nd^/length of flagellomere 14^th^: 2.2×. Ocello-ocular distance/posterior ocelli diameter: 2.3×; distance between posterior ocelli/ocelli diameter: 1.4×.

Mesosoma. Pronotum with two lateral grooves, the lower one excavated. Mesoscutum more or less uniformly sculptured by impressed punctures (distance between punctures about the same as their diameter). Mesoscutum 1.4× wider than long. Mesoscutum and scutellum uniformly covered by dense, pale-coloured pilosity. Scutellum similarly sculptured to mesoscutum. Scutellum length/width at base 1.0×. Scutellar suture broad, with 6-8 costulae. Posterior band of scutellum polished. Scutellar lateral face with polished area less than 30% the face height and about half the face width. Mesopleuron mostly smooth and glabrous, except for punctures on the anterior margin and setae on the all margins; separated from metapleuron by a crenulated sulcus. Metapleuron mostly smooth, with some punctures and setae in the apical half; metapleuron with a crenulate, longitudinal sulcus running from lower margin near metacoxa through spiracle. Metapleural carina raised with a short lamella. Propodeum mostly smooth, with a median carina well defined and raised its entire length; and with a clearly complete transverse carina that reaches the spiracles and forks around them (also with additional, shorter transverse carinae, some of them radiating from the median carina but not reaching the spiracles). Transverse carina on propodeum delimiting two areas, the anterior, basal one being more or less horizontal, while the posterior, apical one is declivous.

Metasoma. Mediotergite 1 mostly smooth and with a deep medial groove over its basal half; slightly widening for the first quarter of its length, then narrowing towards apex; basal width/apical width 2.1×; length/apical width 4.8×. Mediotergite 2 mostly smooth, transverse, subtriangular to trapezoidal in shape; basal width/apical width 0.4×; length/apical width 0.4×. Mediotergite 3 1.5× the length of mediotergite 2. Mediotergite 3 and following unsculptured, polished and with sparse setae. Hypopygium mostly inflexible but with a median, translucid fold ventrally where no pleats are distinguishable. Ovipositor sheaths fully setose, 0.7× as long as metatibia length.

Legs. Metacoxa long, surpassing the length of the third metasomal tergum. Metatibial inner spur 1.6× the length of outer spur, and 0.6× the length of metatarsomere 1. Metafemur 3.2× as long as wide.

Wings. Vein R1a 1.3× as long as stigma length. Stigma 3.1× as long as wide. Length of R1a about 12× as long as the distance between its end and the end of 3RSb. Vein r and 2RS evenly curved to very slightly arched, with no clear limits between the two veins. Vein 2M about the same length of vein (RS+M)b. Edge of vannal lobe of hind wing medially straight to slightly concave and with uniformly distribute setae which are shorter than those at base and apex of the lobe.

Colour: Mostly an orange-yellowish species. Antennal flagellomere and dorsal part of scape brown. Apical edge of scutellum, metascutellum and some carina on propodeum, reddish-brown. Central area on mediotergites 3 and following dark brown. Forewing stigma and most of the wing veins dark brown.

Male. Like the female except for darker coloration as follows: interocellar area, propodeum, metascutellum, apical edge of scutellum, most of the lateral face of scutellum, and most of mediotergites 2+, dark brown to black.

**Figures 1–6.**
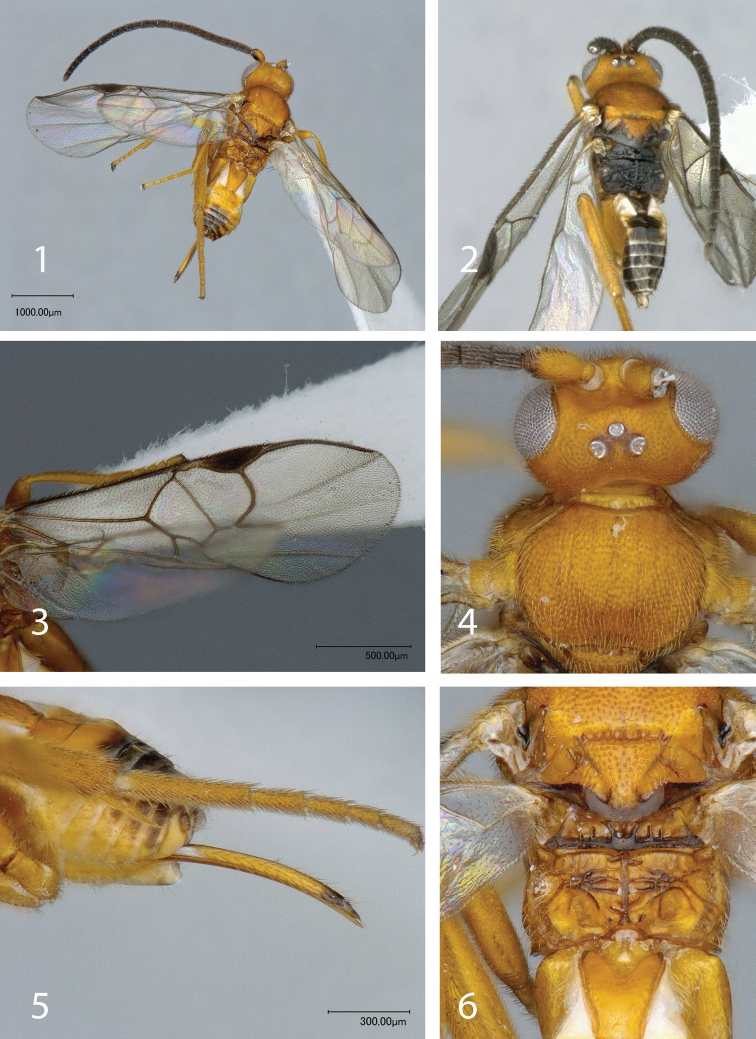
*Mariapanteles felipei* Whitfield. **1** Dorsal habitus, female **2** Dorsal habitus, male **3** forewing, female **4** head and mesoscutum, dorsal view, female **5** hypopygium and ovipositor, lateral view **6** metanotum and propodeum, female, dorsal view.

#### Distribution.

The known specimens were captured in July-August 2007 (full rainy season) by the same Malaise trap placed in old growth rain forest understory on the banks of Rio Francia, where it crosses the access road through Sector Rincon Rain Forest of ACG, at 400 m.

#### Molecular data.

The two known specimens bear the same DNA barcode. The nucleotide sequence in fasta format is:

>*Mariapanteles felipei*

ATTTTATATTTTTTATTTGGAATATGATCTGGAATATTAGGATTTTCATTAAGAATAATTATCCGATTAGAGTTAGGCACACCAGGAAGATTAATTAGAAATGATCAAATCTATAATAGAATTGTTACATCACATGCTTTTATCATAATTTTTTTTATAGTTATACCAATTATAATTGGAGGATTTGGTAATTATTTAATTCCATTAATATTAGCAACTCCTGATATATCATTCCCACGAATAAATAATATGAGATTTTGATTACTAATTCCTTCATTATTTTTATTAATTTTTAGAAGATTTATTAATACAGGAGTAGGTACAGGTTGAACAGTTTATCCACCTTTATCATCAAATTTAGGACATAGAGGTATATCAGTTGATTTAGGAATCTTTTCTCTACATTTAGCAGGAGCCTCATCAATTATAGGAGCAATTAATTTTATTACAACAATTAAAAATATACGAGTTAAATTATTAAAAATAGATAAAATTTCTTTATTTACTTGATCAGTTTTAATTACAGCAATTTTATTATTATTATCTTTACCAGTTTTAGCAGGAGCAATTACTATACTTTTAACAGACCGAAATTTAAATACATCATTTTTTGATCCTTCAGGAGGTGGGGATCCAATTTTATACCAACATTTATTT

#### Etymology.

***Mariapanteles felipei*** is dedicated toLuis Felipe Chavarría Díaz of ACG and San Jose, Costa Rica, in recognition of his 30+ years of dedication to Costa Rican conservation, biodiversity systematics, and biodiversity development throughout Costa Rica, and very specifically within Area de Conservación de Guanacaste.

#### Comments.

The biology of this species, collected with Malaise traps, is unknown. Since its inception in 1978, the ACG caterpillar and parasitoid inventory (Janzen et al. 2009) has achieved Microgastrinae rearings from 9,000+ wild-caught caterpillars and has Malaise-trapped 5,000+ individual Microgastrinae in dry forest, cloud forest and rain forest (Janzen and Hallwachs 2012, Smith et al. 2008); this intense effort has yielded only two conspecific individuals of *Mariapanteles*, both from the same Malaise trap a few weeks apart. While this may suggest that the species is “rare”, it has been the experience of the ACG inventory that when the wasp is finally reared and therefore its host caterpillar known, or the Malaise trap is placed in the “right” place, it may well be found to be common.

### Mariapanteles dapkeyae

Fernández-Triana sp. n.

urn:lsid:zoobank.org:act:EF2F8657-FEBB-4AEE-8501-06B104771B86

http://species-id.net/wiki/Mariapanteles_dapkeyae

Figs 7–12

#### Holotype.

**Female** (CNC).BRAZIL: Mato Grosso, Sinop; x-xi.1975, Malaise Trap; M. Alvarenga col.

#### Paratype.

5 Females and 4 Males (CNC, with 1 female each deposited in INHS and BMNH). Same data as for holotype, except for collecting date (x.1974 for all specimens but two males with collecting date: x.1975). Two males deposited in the CNC have DNA Voucher codes: CNCHYM 03387 and CNCHYM 07145. 1 Female (CNC). BRAZIL: Goiás, Jatai; xi.1972; F. M. Oliveira col.

#### Description.

**Female.** Antenna about the same length as body; body length 2.4 mm; forewing 2.6 mm. Head. Face with shallow and sparse punctures and sparse, uniformly distributed setae. Face width at antennal base/face width at clypeus edge: 1.1×; intertentorial pit distance/face width at clypeus edge: 0.4×; compound eye height/head height: 0.8×; head height/width: 0.8×; face width at antennal base/head maximum width: 0.5×; malar space/basal width of mandible 1.4×; clypeus width/height: 3.5×. Length/width of flagellomeres: 2nd (2.4×), 8th (2.5×), 14th (1.3×). Length of flagellomere 2^nd^/length of flagellomere 14^th^: 2.2×. Ocello-ocular distance/posterior ocelli diameter: 2.2×; distance between posterior ocelli/ocelli diameter: 1.3×.

Mesosoma. Pronotum with two lateral grooves present, the lower one excavated. Mesoscutum more or less uniformly sculptured by shallowly impressed punctures (distance between punctures about the same as their diameter). Mesoscutum 1.3× wider than long. Mesoscutum and scutellum uniformly covered by dense, pale yellow pilosity. Scutellum mostly smooth, with very shallow and sparse punctures. Scutellum length/width at base 1.1×. Scutellar suture broad, with 4-6 costulae. Posterior band of scutellum polished. Scutellar lateral face with polished area less than 20% the face height and less than half the face width. Mesopleuron mostly smooth and glabrous, except for punctures on the anterior margin and setae on all margins; separated from metapleuron by crenulate sulcus. Metapleuron mostly smooth, with some punctures and setae in the apical half; metapleuron with a crenulated, longitudinal sulcus running from lower margin near metacoxa through spiracle. Metapleural carina raised with a short lamella. Propodeum mostly smooth; with a median carina well defined and raised its entire length; and with a clearly complete transverse carina that reaches the spiracles and forks around them (there are also additional, shorter transverse carinae). Transverse carina on propodeum delimiting two areas, the anterior, basal one that is more or less horizontal, and the posterior, apical one is declivous.

Metasoma. Mediotergite 1 mostly smooth and with a deep medial groove over its basal half; slightly widening for the first quarter of its length, then narrowing towards apex; basal width/apical width 1.5×; length/apical width 3.3×. Mediotergite 2 mostly smooth, transverse, subtriangular to trapezoidal in shape; basal width/apical width 0.3×; length/apical width 0.4×. Mediotergite 3 1.5× the length of mediotergite 2. Mediotergite 3 and following unsculptured, polished and with sparse setae. Hypopygium mostly inflexible but with a median, translucid fold ventrally where 1–2 weak pleats are sometimes distinguishable. Ovipositor sheaths fully setose, 0.7× as long as metatibia length.

Legs. Metacoxa long, surpassing the length of the third metasomal tergum. Metatibial inner spur 1.4× the length of outer spur, and 0.5X the length of metatarsomere 1. Metafemur 3.2× as long as wide.

Wings. Vein R1a 1.3× as long as stigma length. Stigma 3.3× as long as wide. Length of R1a about 14× as long as the distance between its end and the end of 3RSb. Vein r and 2RS evenly curved to very slightly arched, with no clear limits between the two veins. Vein 2M about the same length of vein (RS+M)b. Edge of vannal lobe of hind wing medially straight to slightly concave and with uniformly distribute setae which are shorter than those at base and apex of the lobe.

Colour: Mostly yellow, with antennal flagellomere, forewing stigma and most of the wing veins, light brown.

Male. Mostly like females, but some specimens with darker interocellar area and mediotergites 3+. We associate these males with these females because of their morphological similarity.

**Figures 7–12.**
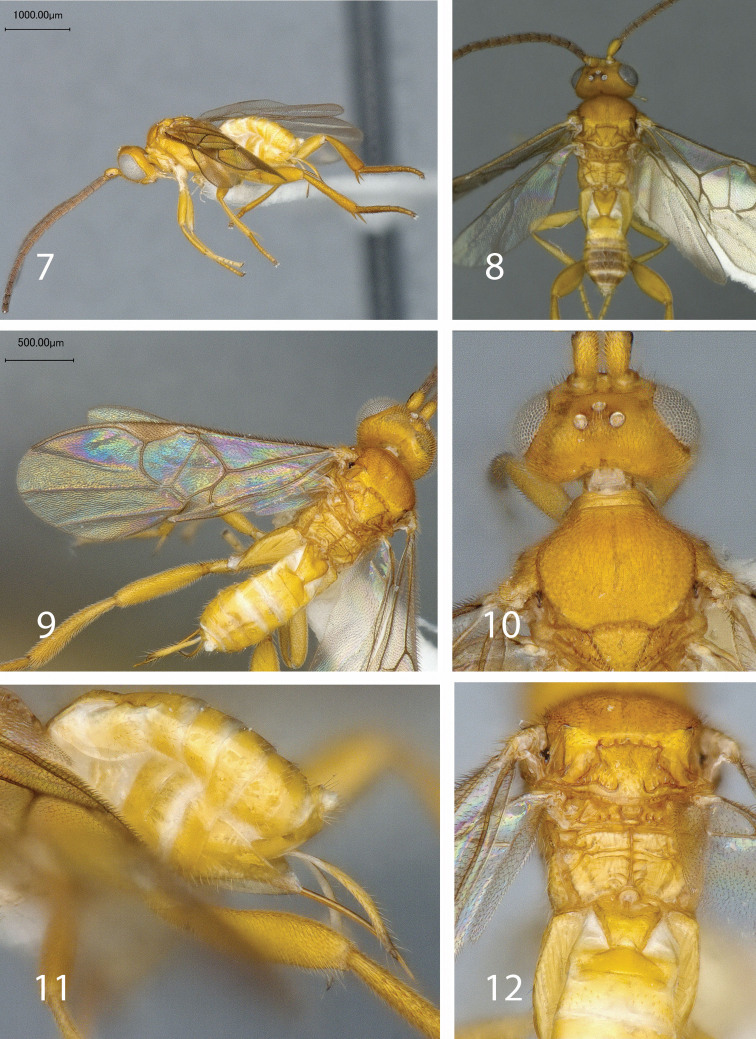
*Mariapanteles dapkeyae* Fernandez-Triana. **7** lateral habitus, female **8** dorsal habitus, female **9** fore wing, female **10** head and mesoscutum, dorsal view, female **11** hypopygium and ovipositor, lateral view **12** metanotum, propodeum and anterior metasomal tergites, female, dorsal view.

#### Distribution.

The specimens were collected with Malaise traps in two Brazilian localities (less than 1000 km apart) which are presumed to have been rain forests at the time of collecting.

#### Molecular data.

Two paratype male specimens rendered partial barcodes (361 bp for the one with DNA voucher code CNCHYM 03387, and 164 bp for the one with DNA voucher code CNCHYM 07145). The nucleotide sequence shown below corresponds to the longer sequence in the barcode region of COI:

>*Mariapanteles dapkeyae*

TAAGATTTTGATTATTAATTCCATCTTTATTTATATTAATTTTTAGAAGATTTATTAATACAGGAGTAGGTACAGGTTGAACAGTATACCCACCATTATCATCAAATTTAAGACATAGGGGCATATCAGTCGATTTAAGAATTTTTTCTTTACATTTAGCAGGAACTTCATCAATTATAGGAGCAATTAATTTTATTACAACAATTAAAAATATACGAGTTAAATTATTTAAAATAAATAAAATTTCTTTATTTAATTGATCAGTTTTAATTACAGCAATTTTATTATTATTATCATTACCAGTATTAGCAGGTGCTATTACTATACTTTTAACAGATCGAAATTTAAATACATCATTTTT

#### Etymology.

This species is dedicated to Tanya Heckmann Dapkey of Philadelphia, Pennsylvania, USA, in recognition of her seven years of diligent and highly accurate sorting, processing, databasing, and de-legging ACG microgastrine wasps for DNA barcoding.

#### Comments.

The biology of this species, collected with Malaise traps, is unknown. In the CNC collection there are two additional specimens of *Mariapanteles* from Brazil: one female from Piedra Azul, Minas Gerais; and one male from Rio Javari, Estirar do Equador, Amazonas. Both specimens differ morphologically from *Mariapanteles dapkeyae*. Additionally, the male specimen (with DNA voucher code CNCHYM 03380) rendered a partial DNA barcode (164bp) which has 7 base pairs different (4.3 %) from the barcoded specimens of *Mariapanteles dapkeyae*. We believe those two specimens may represent additional species, but because they are singletons we have not described them as new species here.

**Figure 13.**
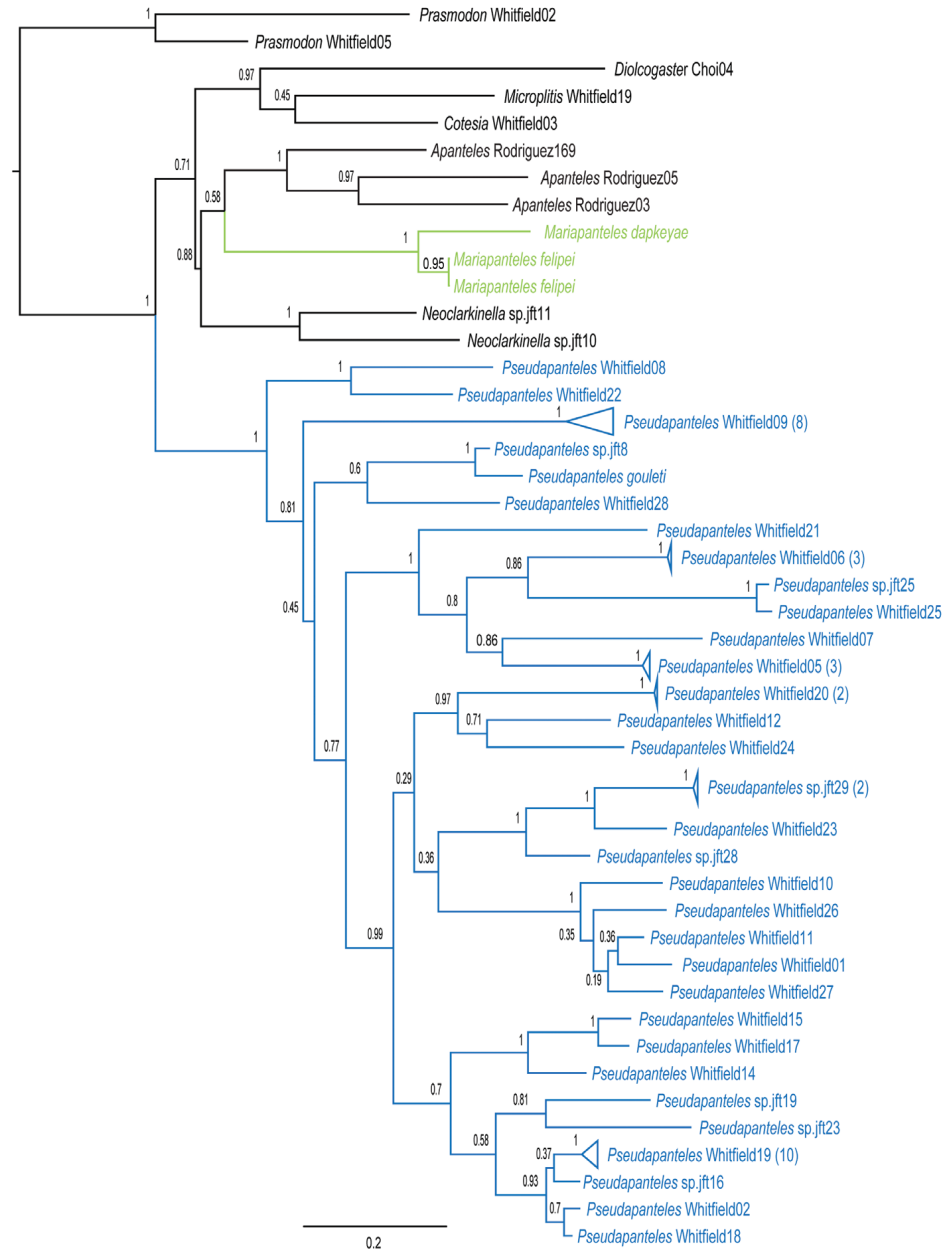
Maximum clade credibility tree for *Pseudapanteles* and *Mariapanteles* based on Bayesian analysis of COI sequences, with 3^rd^ codon positions included (see text for details). Values at nodes are posterior probabilities.

**Figure 14.**
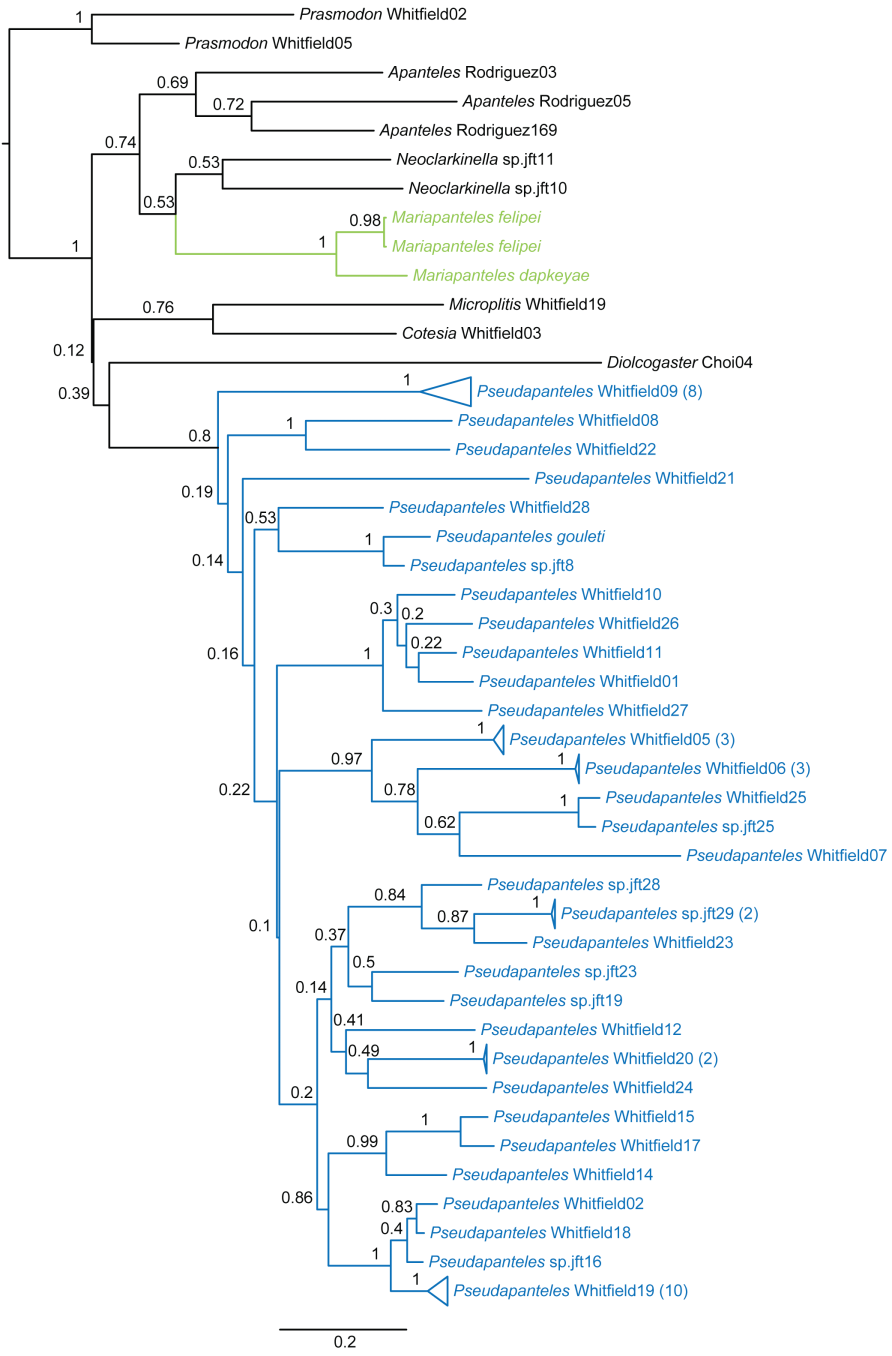
Maximum clade credibility tree for *Pseudapanteles* and *Mariapanteles* based on Bayesian analysis of COI sequences, with 3^rd^ codon positions excluded (see text for details). Values at nodes are posterior probabilities.

## Supplementary Material

XML Treatment for Mariapanteles

XML Treatment for Mariapanteles felipei

XML Treatment for Mariapanteles dapkeyae

## Appendix 1

Detailed information about collection, BOLD, and GenBank number of species. (doi: 10.3897/zookeys.208.3326.app) File format: Excel spreadsheet (xls).

**Explanation note:** Appendix I contains all available DNA barcodes for Pseudapanteles (32 New World species, most of which are undescribed) and Mariapanteles (2 species) that were at least 300 bp long were downloaded from BOLD.
